# Leaf drought and heat tolerance are integrated across three temperate biome types

**DOI:** 10.1038/s41598-025-95623-5

**Published:** 2025-04-09

**Authors:** Denise Mitchell, Leonie Schönbeck, Shukan Shah, Louis S. Santiago

**Affiliations:** 1 Department of Botany & Plant Sciences, University of California, 2150 Batchelor Hall, Riverside, CA 92521 USA; 2 Southern Swedish Forest Research Center, Swedish University of Agricultural Sciences, Alnarp, Sweden; 3 Smithsonian Tropical Research Institute, Ancon, Balboa, Panama

**Keywords:** Drought, Cellular electrolyte leakage, Leaf turgor loss, Photosynthesis, Thermotolerance, Ecophysiology, Plant physiology

## Abstract

Leaf-scale heat and drought tolerance provide direct measures of the ability to withstand environmental stress and can be used to evaluate plant susceptibility to emerging climatic extremes. However, recent droughts increasingly occur with heatwaves, causing plants to withstand two simultaneous environmental stresses. Tolerance of leaf-level processes to heat and drought stress have mostly been studied independently, preventing an understanding of whether tolerance co-occurs for these two environmental stresses. To address this, we measured leaf photosynthetic heat tolerance as the critical temperatures at which photosystem II efficiency starts to decrease (T_crit_) and shows a decrease of 50% (T_50_) or 95% (T_95_) in three temperate biomes (desert, oak-pine forest, and mediterranean-type shrubland). We also characterized drought tolerance as the water potential at leaf turgor loss point (π_tlp_) and cellular membrane stability in response to simulated drought. We found coordination of heat and drought tolerance through a significant relationship of π_tlp_ with T_50_ and T_crit_ that varied with season, whereas T_95_ showed no relation to π_tlp_. Species with greater drought tolerance also showed greater membrane stability, implicating membrane leakiness as a potential mechanism of physiological decline during stress. Despite local variation in temperature and precipitation extremes, leaf heat and drought tolerance converged to common cross-biome relationships, providing evidence of interdependence that spanned distinct climates.

**Supplementary Information:**

The online version contains supplementary material available at 10.1038/s41598-025-95623-5.

## Introduction

Climate-change type drought is linked to vegetation mortality events around the world^1,2^. However, the extended droughts that are characteristic of recent climate change increasingly co-occur with extreme temperatures, leading to combined drought and heatwave events that are compounded by low background aridity and persistent dry conditions^3^. These emerging climatic patterns cause plants to withstand two environmental stresses that simultaneously impose limits to plant productivity, but through multiple varying mechanisms. Tools for characterizing a broad range of plant species for their drought and heat tolerance are achieving sustained focus due to the need to understand the mechanisms that determine which species are most susceptible to mortality and how climatic alterations will shape future plant communities. Currently, developing predictive tools to incorporate plant mortality responses into dynamic vegetation models is the biggest challenge for understanding the climate change feedback of plant mortality to the climate-carbon system^4,5^.

Much of the attention on plant drought and heat tolerance is directed at leaves because leaves are the primary source of photosynthetic productivity. The water potential at leaf turgor loss point (π_tlp_) is a chief parameter for characterizing relative drought tolerance among species and signifies the point at which leaf cells lose turgor, or wilt^6^. While this does not determine plant mortality, it is highly correlated with the suite of plant traits that explain relative drought survival among co-occurring plant species^7^, and is therefore measured broadly across plant species as a basis for comparative drought tolerance. The temperatures at which photosystem II efficiency starts to decrease (T_crit_) and shows a decrease of 50% (T_50_) or 95% (T_95_), have emerged as principal parameters for characterizing comparative leaf photosynthetic heat tolerance^8,9^. There is some data^10,11^, and more theory, suggesting that plants resist many stresses through the same mechanisms. The Integrated Response of Plants to Stress concept suggests that plants respond similarly to a variety of stresses, including water deficit, nutrient deficiency and heavy metal toxicity, by closing stomata, suspending reproduction, and diverting recent photosynthate away from growth and towards storage^12^. Considering trends in compound drought and heatwave events, it is notable that these two leaf-scale indices of stress tolerance have the potential to elucidate the degree of cross-tolerance to heat and drought^11^.

Here, we measured π_tlp_, T_crit_, T_50_, and T_95_, in 21 woody plant species in desert, forest, and Mediterranean-type shrubland biomes across two seasons (Tables S1, S2). While π_tlp_, T_crit_, T_50_, and T_95_, have emerged as important climate tolerance indices, it is also important to note that plasticity in these parameters can occur due to leaf exposure to hot, cold, or dry seasons^13–15^. Values for π_tlp_ vary between pre-drought and post-drought conditions, with pre-drought π_tlp_ having a major impact on post-drought π_tlp_^16^. In addition, T_crit_ and T_50_have been shown to increase in the dry season in association with reduced leaf relative water content and increased leaf temperatures^15^. Within our study system, leaf production occurs between April and early June, and peak summer temperatures occur in August and September. Therefore, we conducted measurements early in the season (May-June; Early Season), representing relatively newly mature leaves that had not been exposed to seasonal drought or maximum seasonal temperatures, and late in the season (December-March; Late Season), representing leaves post-heat and post-drought. We determined correlations between π_tlp_, T_crit_, T_50_, and T_95_ as diagnostics of plant capacity to withstand environmental extremes.

Because the membrane bound photosystem II and D1 protein are considered the most thermally labile components of photosynthesis^17,18^, we also tested whether leaf cellular membrane stability under drought conditions is related to drought resistance among a select group of Mediterranean-type shrubs under controlled growing house conditions to evaluate a possible mechanism that interlinks these environmental resistances. Our hypothesis was that leaf heat and drought tolerance are related because the effects of heat and drought converge on membranes, and our research was guided by the following questions: (1) Are leaf-scale drought and heat tolerance related across plant species from three temperate biomes? (2) Does the seasonal timing of measurement in relation to peak heat and drought change the relationship between heat and drought tolerance? (3) Is there evidence that leaf cellular membrane stability during stress is involved in environmental tolerance?

## Results and discussion

**Fig. 1:**
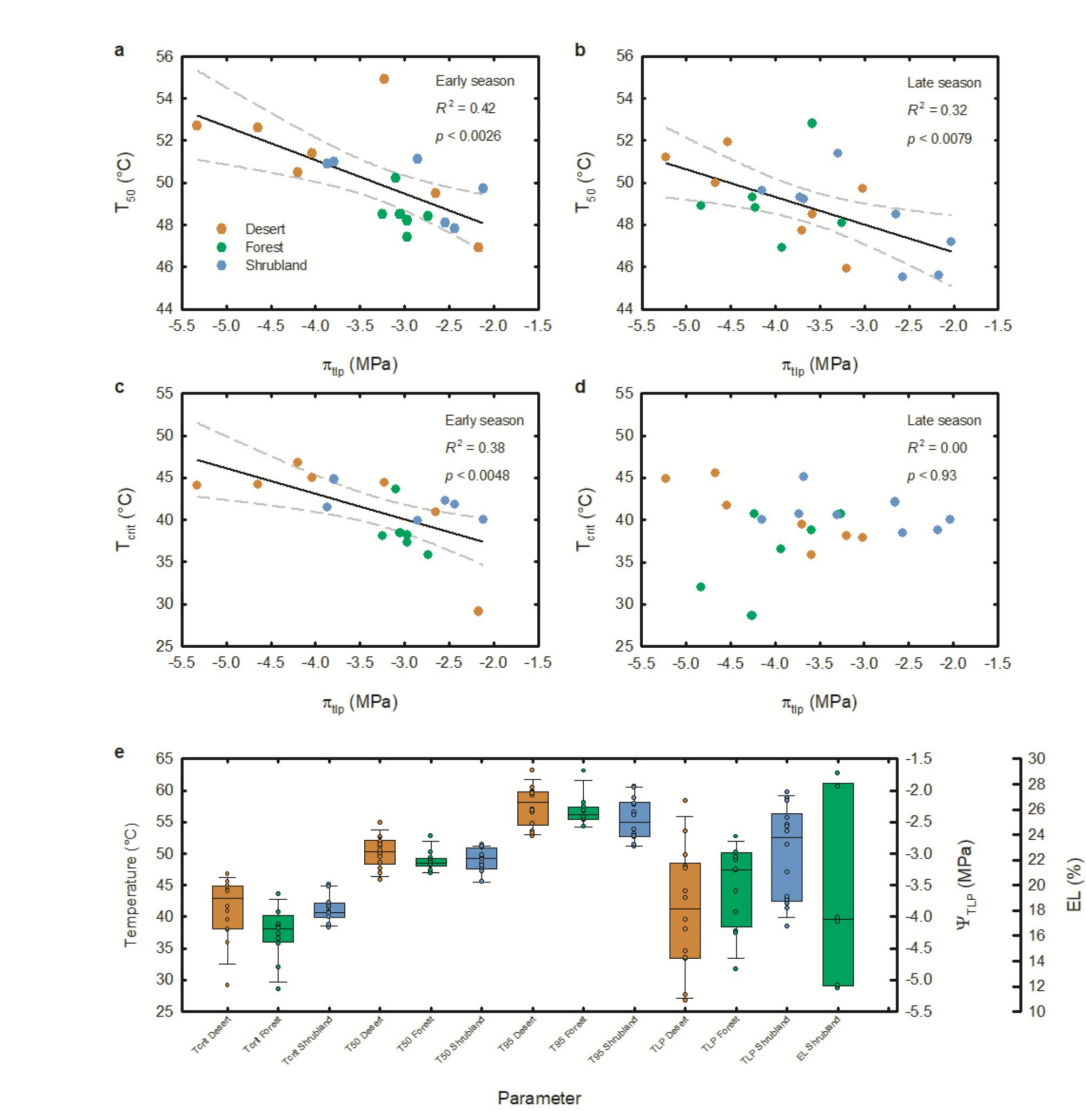
Relationship between leaf thermotolerance and drought resistance for 21 woody plant species from three biomes: Desert (brown); Temperate Forest (green); Shrubland (blue). (**a**) Early season leaf temperature at 50% loss of chlorophyll fluorescence value (T_50_) as a function of leaf water potential at turgor loss point (π_tlp_); (**b**) Late season T_50_ as a function of π_tlp_; (**c**) Early season critical leaf temperature at which chlorophyll fluorescence value begins to decline (T_crit_) as a function of π_tlp_; (**d**) Late season T_crit_ as a function of π_tlp_ ; (**e**) Box plots of parameter values for thermotolerance parameters, turgor loss point (TLP), and electrolyte leakage (EL) with error bars showing the 5th and 95th percentile, the top and bottom of the box are the 25th and 75th percentiles, the line inside the box is the median. Points show individual measurements of study species. a-c) Solid black lines indicate best-fit regression lines and dashed grey lines indicate 95% confidence intervals for linear regression.

Regression analysis showed a significant negative relationship of π_tlp_ with T_50_ and with T_crit_ early in the season, before peak heat and seasonal drought, and only between π_tlp_ and T_50_ late in the season, post-heat and post-drought, whereas T_95_ showed no relation to π_tlp_ (Fig. 1; Table 1). These results indicate that species with greater drought tolerance also withstood higher temperatures before photosynthetic processes began to decline. Relationships of π_tlp_ with T_50_ and with T_crit_ only occurred when compared across all biomes but not within a specific biome, likely due to small sample sizes within each biome (Table 1). This represents the first study to compare these two environmental stress tolerance indices among species from multiple biomes, and this finding is consistent with theory predicting an integrated response of plants to stress^12^, and with previous evaluations of plant stress interactions such as drought and nutrient limitation^19,20^.

**Table 1:** Results of standard mean axis regression analysis for relationships between drought tolerance expressed as water potential at leaf turgor loss point (π_tlp_), versus the temperatures at which photosystem II efficiency starts to decrease (T_crit_) and shows a decrease of 50% (T_50_) or 95% (T_95_). Results are shown within each biome, and across all biomes.

	Slope	Intercept	*r* ^2^	*p*-value	Heterogeneity of slope	Heterogeneity of intercept	Common Slope
Model: T_crit_ = *m* ⋅ π_tlp_ + *b*							
*Early Season*							
Desert	−5.3 (−11.1, −2.5)	22.1 (5.2, 38.9)	0.5	0.07			
Forest	−15.3 (−42.1, −5.6)	−7.6 (−62.6, 47.4)	0.26	0.3			
Shrubland	−2.5 (−6.5, −0.9)	34.5 (26.0, 43.0)	0.32	0.24			
All	−4.9 (−7.2, −3.3)	24.9 (18.3, 31.6)	**0.38**	**0.005**	**0.04**		
*Late Season*							
Desert	−4.5 (−7.5, −2.6)	22.7 (12.9, 32.6)	**0.78**	**0.09**			
Forest	9.0 (3.6, 22.6)	72.2 (33.9, 110.7)	0.42	0.17			
Shrubland	−2.7 (−6.0, −1.2)	32.6 (24.9,40.2)	0.18	0.3			
All	−4.7 (−7.4, −2.9)	22.4 (13.8, 31.0)	0	0.93	0.13	**0.005**	
Model: T_50_ = *m* ⋅ π_tlp_ + *b*							
*Early Season*							
Desert	−2.3 (−5.3, −1.0)	42.6 (34.2, 51.0)	0.34	0.17			
Forest	−5.3 (−15.8, −1.8)	32.6 (11.3, 53.8)	0.09	0.56			
Shrubland	−2.0 (−5.0, −0.8)	43.8 (37.4, 50.1)	0.45	0.14			
All	−2.5 (−3.6, −1.7)	41.9 (38.7, 45.1)	**0.42**	**0.003**	0.33	0.2	0.09
*Late Season*							
Desert	−2.5 (−5.2, −1.2)	39.3 (31.1, 47.5)	0.51	0.0718			
Forest	3.6 (1.2, 11.0)	63.6 (43.5, 83.6)	0.02	0.78			
Shrubland	−2.6 (−5.0, −1.4)	40.4 (34.8, 46.0)	**0.53**	**0.04**			
All	−2.3 (−3.4, −1.6)	40.4 (36.9, 43.9)	**0.32**	**0.008**	0.84	0.24	0.15
Model: T_95_ = *m* ⋅ π_tlp_ + *b*							
*Early Season*							
Desert	2.8 (1.0, 7.4)	68.8 (56.2, 81.5)	0.01	0.81			
Forest	−6.6 (−20.6, −2.1)	36.4 (8.6, 64.2)	0.002	0.94			
Shrubland	−4.4 (−13.0, −1.5)	43.7 (26.2, 61.2)	0.1	0.55			
All	−3.2 (−5.2, −2.0)	46.8 (41.3, 52.3)	0.04	0.42	0.48	0.89	0.1
*Late Season*							
Desert	4.0 (1.4, 10.1)	71.9 (53.9, 89.9)	0.002	0.93			
Forest	−5.8 (−17.8, −1.9)	33.5 (1.1, 66.0)	0.04	0.72			
Shrubland	−4.3 (−8.0, −2.1)	41.7 (31.1, 52.2)	0.37	0.11			
All	−3.8 (−5.9, −2.5)	42.0 (35.7, 48.4)	0.17	0.06	0.82	0.59	0.08

The relationship between π_tlp_ and T_crit_ varied between seasons, with significant relationships between π_tlp_ and T_50_ in both seasons and a stronger relationship in the early season (*r*^2^ = 0.42, *p* = 0.003; Fig. 1a) than in the late season (*r*^2^ = 0.32, *p* = 0.008; Fig. 1b), and with a similar magnitude for the early season π_tlp_ and T_crit_ relationship (*r*^2^ = 0.38, *p* = 0.005; Fig. 1c), but no significant relationship in the late season for π_tlp_ and T_crit_ (Fig. 1d; Table 1). Peak heat and drought caused an increase in drought tolerance, with a significant mean reduction in π_tlp_ of 0.4 MPa from the early to the late season (95% CI −0.66, −1.3MPa), consistent with studies showing a seasonal ability for acclimation to dry conditions^13,14,16^. Peak heat and drought also caused significant decreases in heat tolerance, with a mean reduction in 1.6 ºC in T_crit_ (95% CI −0.08, −3.25 MPa) and 1.1 ºC in T_50_ (95%. CI −0.24, −1.91 MPa), but no significant difference in T_95_. The result that plants became more drought tolerant but less heat tolerant after peak heat and drought further explains the weakening or absence of significance in late season π_tlp_-thermotolerance relationships. Lower heat tolerance in the late season, when temperatures were cooler is also consistent with studies showing acclimation of T_50 _to ambient conditions^15,21,22^. Interestingly, T_95_ was not related to π_tlp_ in either season and our estimates of T_95_ are often greater than our highest incubation temperature of 54 °C, suggesting that extrapolating beyond the range of fit in estimating T_95_ may have contributed to more variation and possibly more uncontrolled error in estimates of leaf thermotolerance near the limit of function.

**Fig. 2:**
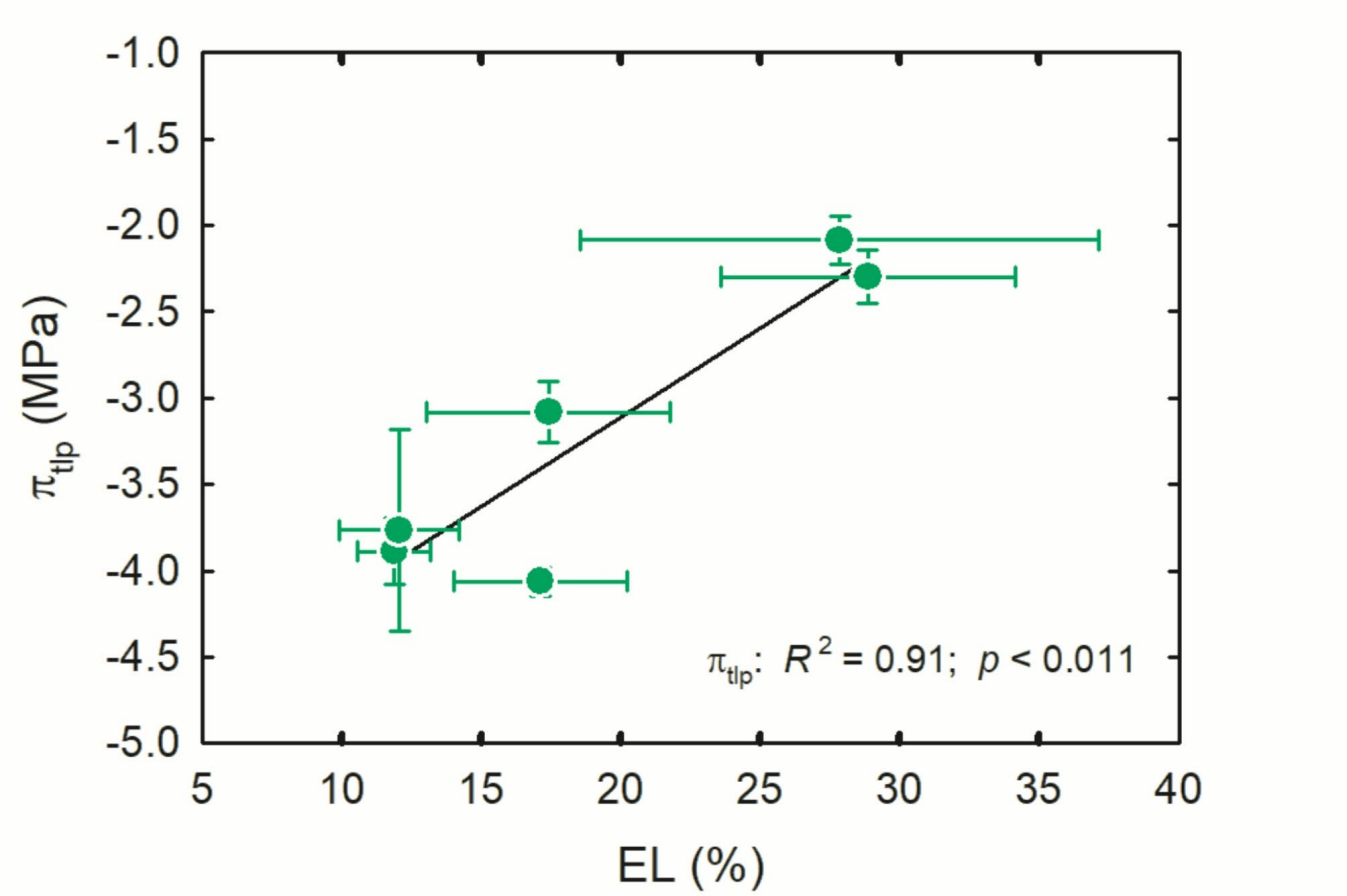
Leaf water potential at turgor loss point (π_tlp_) as a function of membrane stability measured as cellular electrolyte leakage (EL) determined on six species of shrubland plants growing in well-watered controlled greenhouse conditions. Each point is the mean of a species; Error bars represent ± 1SE. Solid black line indicates best-fit regression line for π_tlp_.

Leaf cellular membrane stability determined by electrolyte leakage was related to more resistant π_tlp_ (Fig. 2), implicating membrane integrity as a mechanism associated with maintenance of leaf drought tolerance. Our analysis of leaf cellular membrane stability is consistent with the idea that membrane damage occurs during low cellular water potential conditions^23^. Plants under both high temperature and drought stress respond by remodeling membrane fluidity and releasing α-linolenic (18:3) from membrane lipids^24^. These responses are maximized in the chloroplast, where drought stress causes lipolytic and peroxidative activities that decrease membrane lipid content, and high temperatures cause denaturation of photosynthetic proteins in chloroplast membranes^23,24^. Thus, high temperatures and drought stress converge to loosen and denature membranes, and represent a common hazard to cellular integrity and function during environmental extremes. Such responses are now known to be linked to crosstalk between signaling compounds, hormones and mitogen-activated protein kinases (MAPKs) that connect plant responses to multiple environmental stresses^25^, with changes in lipid structure in response to osmotic stress regulated by mitogen-activated protein kinase 6 (MPK6)^23^.

Whereas the same relationship that we found between leaf-scale heat and drought tolerance has been shown once, in one site of a temperate forest^11^, we show that despite local variation in temperature and precipitation extremes across sites, leaf heat and drought tolerance converge to a common cross-biome relationship, illustrating integrated environmental tolerances that span major global biome-types. However, leaf temperatures are another important part of this stress assessment. The limited homeothermy hypothesis suggests that leaves thermoregulate through transpiration to maintain leaf temperatures near optimal values for photosynthesis^26^. Yet, recent empirical work in North and Central America show that canopy leaves are warmer than air during most of the day, including during the majority of ecosystem photosynthesis^27^. Therefore, concurrent leaf and air temperatures would need to be incorporated into future efforts by evaluating the realized leaf temperatures and thermal safety margins. Our findings contribute to understanding leaf thermotolerance by providing evidence that leaf responses to heat and drought are coordinated, and we identify integration as an important component of future models that predict plant responses to compound drought and heatwave events^2,3,28^. Such findings open the possibility of ranking species in terms of their ability to withstand combined stress and facilitating predictions of future community composition based on relative positionality along a hierarchy of stress tolerance.

Overall, our results are of twofold interest: (1) they demonstrate that plant responses to two of the primary environmental stresses causing plant mortality during climate change show a common coordination across three biomes, and (2) they suggest that including coordinated heat and drought tolerance can improve model predictions of the responses of natural vegetation to climatic extremes to better forecast hotter and drier climate change-induced plant mortality events.

## Methods

### Study sites

 Leaf samples were collected from three sites, representing major temperate biomes (Sonoran Desert, Temperate Oak-Pine Forest, and Mediterranean-type Shrubland; Table S1). Leaf production occurs between April and early June, and peak summer temperatures occur in August and September. Therefore, samples were collected across two seasons: 1) May-June (Early), representing relatively newly mature leaves that had not been exposed to seasonal drought or maximum seasonal temperatures, and December-March (Late), representing leaves post-heat and post-drought. Newly mature leaves for heat and drought tolerance measurements were collected from 3 to 5 individuals early (08:00–10:00 h), sealed in plastic bags and stored in coolers until transported to the laboratory within 2 h. Species selection represents the 6–8 most common species at each site (Table S2). Sample collection occurred between January 2020 – December 2021 (Table S3)^29^.

### Drought tolerance

Leaf water potential at turgor loss (π_tlp_) was measured as cellular osmotic potential^7^. Three leaf discs (6.2 mm diameter) were excised from leaves of three individuals, wrapped in aluminum foil and submerged in liquid N_2_ for 2 min to rupture cellular structure, then placed in a 5600 vapor pressure osmometer (Wescor) for measurement of osmolality (mOsm kg^−1^). Upon removal from liquid N_2_, leaf discs were punctured ten times with sharp-tipped forceps before sealing in the osmometer chamber. Final osmometer readings were converted to π_osm_ using the van’t Hoff Eqs^7,30^.

### Heat tolerance

 Heat tolerance was assessed using chlorophyll *a *fluorescence^8,9^. Leaf discs (10 mm diameter) were excised between major veins. In the case of needles, the disc consisted of 3–5 needle segments. Discs were placed in sealable plastic bags, and immersed in a preheated water bath for 15 min using 8–11 incubation temperatures between 38 and 54 °C, depending on biome (Table S4). Subsequent to heat exposure, discs were dark-acclimated for 15 min before measurement of relative chlorophyll *a* fluorescence (*F*_v_/*F*_m_) with a pulse-amplitude modulated (PAM) chlorophyll fluorometer (Walz).

#### Membrane stability

 Leaf cellular electrolyte leakage in response to simulated water stress was measured on a subset of six species that occur at the mediterranean-type shrubland site (*Ceanothus tomentosus*, *Heteromeles arbutifolia*, *Malosma laurina*, *Quercus berberidifolia*, *Salvia apiana*, *Salvia mellifera*) growing under well-watered greenhouse conditions. Three mature leaves from three individuals were collected between 08:00–09:00 h and wiped to remove debris. One leaf disc (10 mm diameter) from each leaf was excised and immediately submerged in a hypertonic solution (−6.5MPa) of 3350 Polyethylene glycol, for 8 h, then rinsed and placed in distilled water, measured for initial values of electrical conductivity (CE) with a 6 + meter (Orion), and allowed to soak for 16 h before subsequent CE measurement. Samples were then boiled for 20 min and measured for total CE 8 h later. Electrolyte leakage minus initial CE was expressed as a fraction of total CE^31^.

#### Statistical analysis

 Leaf heat and drought tolerance variables were normally distributed. Bivariate relationships between leaf heat and drought tolerance parameters were tested using standardized major axis (SMA) estimation using the ‘smatr’ package in R Statistical Software. T_crit_, T_50_, and T_95_ were modeled based on the relationship of *F*_v_/*F*_m_ versus temperature for each species with the ‘nls’ function in R [nls(*F*_v_/*F*_m_ ~ θ_1_/(1 + exp(–(θ_2_ + θ_3_ × Temperature)))] where θ_1_ is the control value of *F*_v_/*F*_m_ (≈ 0.8) and θ_2_ and θ_3_ are the intercept and slope coefficients of the logit(*F*_v_/*F*_m_) ~ Temperature relationship, respectively^32,33^), with bootstrapped means calculated by randomly resampling data and fitting a new model for each species 1000 times. Here, T_crit_ was defined as the temperature at which *F*_v_/*F*_m_ begins to decline, calculated as the temperature at which the slope of the *F*_v_/*F*_m_versus temperature reached 15% of its most negative value^32^.

## Electronic supplementary material

Below is the link to the electronic supplementary material.

Supplementary Material 1

## Data Availability

Data used for this study is available at Figshare (https://figshare.com/).
