# Supplementary material for: Leaf drought and heat tolerance are integrated across three temperate biome types
Source: Sci Rep. 2025 Apr 9;15:12201. doi: 10.1038/s41598-025-95623-5 (PMC11982534; doi:10.1038/s41598-025-95623-5)
Supplement: Supplementary file 1 — Supplementary Material 1 [file 41598_2025_95623_MOESM1_ESM.docx]

Supplementary Materials

**Table S1.** Study site characteristics for three sites in contrasting biomes, including mean annual precipitation (MAP), mean annual temperature (MAT) and elevation.

| Biome | MAP (mm) | MAT (°C) | Elevation (m) | Site location |
| --- | --- | --- | --- | --- |
| Desert | 139 | 23.3 | 275 | Boyd Deep Canyon Desert Research Center |
| Forest | 637 | 12.6 | 1770 | James San Jacinto Mountains Reserve |
| Shrubland | 360 | 16.4 | 150 | Santa Margarita Ecological Reserve |

**Table S2.** List of species studied with respective plant taxonomic family, biome, and leaf growth habit.

| **Species** | **Family** | **Biome** | **Leaf Growth Habit** |
| --- | --- | --- | --- |
| *Condea emoryi* | Lamiaceae | Desert | Semi Deciduous |
| *Encelia farinosa* | Asteraceae | Desert | Evergreen |
| *Fouquieria splendens* | Fouquieriaceae | Desert | Drought Deciduous |
| *Justicia californica* | Acanthaceae | Desert | Drought Deciduous |
| *Larrea tridentata* | Zygophyllaceae | Desert | Evergreen |
| *Parkinsonia florida* | Fabaceae | Desert | Semi Deciduous |
| *Senegalia greggi* | Fabaceae | Desert | Semi Deciduous |
| *Abies concolor* | Pinaceae | Forest | Evergreen |
| *Arctostaphylos pringlei* | Ericaceae | Forest | Evergreen |
| *Calocedrus decurrens* | Cupressaceae | Forest | Evergreen |
| *Pinus coulteri* | Pinaceae | Forest | Evergreen |
| *Pinus lambertiana* | Pinaceae | Forest | Evergreen |
| *Quercus chrysolepis* | Fagaceae | Forest | Evergreen |
| *Adenostoma fasciculatum* | Rosaceae | Shrubland | Evergreen |
| *Artemisia californica* | Asteraceae | Shrubland | Drought Deciduous |
| *Eriogonum fasciculatum* | Polygonaceae | Shrubland | Evergreen |
| *Heteromeles arbutifolia* | Rosaceae | Shrubland | Evergreen |
| *Malosma laurina* | Anacardiaceae | Shrubland | Evergreen |
| *Rhamnus illicifolia* | Rhamnaceae | Shrubland | Evergreen |
| *Salvia apiana* | Lamiaceae | Shrubland | Semi Deciduous |
| *Salvia mellifera* | Lamiaceae | Shrubland | Semi Deciduous |

**Table S3.** Site-based climate data during leaf sampling. Mean and maximum temperatures and total precipitation for each year the study was conducted.

| Temperature |  |  |  |
| --- | --- | --- | --- |
| Biome | Year | Mean (°C) | Max (°C) |
| Desert*^a^* | 2020 | 25.4 | 43.1 |
|  | 2021 | 25.3 | 41.2 |
|  |  |  |  |
| Forest*^b^* | 2020 | 10.7 | 36.3 |
|  | 2021 | 10.6 | 36.8 |
|  |  |  |  |
| Shrubland*^c^* | 2020 | 18.4 | 44.2 |
|  | 2021 | 17.7 | 39.1 |
|  |  |  |  |
| Precipitation |  |  |  |
| Biome | Year | Total (mm) |  |
| Desert*^a^* | 2020 | 112.8 |  |
|  | 2021 | 78.5 |  |
|  |  |  |  |
| Forest*^b^* | 2020 | 524.3 |  |
|  | 2021 | 540.0 |  |
|  |  |  |  |
| Shrubland*^c^* | 2020 | 406.4 |  |
|  | 2021 | 289.1 |  |

*^a^*https://deepcanyon.ucnrs.org/weather-data/

*^b^*https://james.ucnrs.org/weather-data/

*^c^*https://fsp.sdsu.edu/cameras-and-weather-station/

**Fig. S4.** Incubation Temperatures for leaf thermotolerance assays.

| Desert | Forest | Shrubland Early | Shrubland Late |
| --- | --- | --- | --- |
| Temperature | (°C) |  |  |
| 0 | 0 | 0 | 0 |
| 38 | 38 | 38 | 40 |
| 40 | 42 | 40 | 42 |
| 42 | 44 | 42 | 44 |
| 44 | 46 | 44 | 46 |
| 46 | 48 | 46 | 48 |
| 48 | 50 | 48 | 50 |
| 50 | 52 | 50 | 52 |
| 52 |  | 52 |  |
| 54 |  | 54 |  |
| 56 |  |  |  |
